# From supply to strategy: leveraging central medical stores experiences and information for health system strengthening

**DOI:** 10.1080/20523211.2026.2649302

**Published:** 2026-03-30

**Authors:** Maria-Belen Tarrafeta-Sayas, Christophe Rerat, Sophie Pilon, Mieja Vola Rakotonarivo, Louis Dèhoumon Koukpemedji, Serge Yapo, Rachel Duncan, Danielle Hodjo, Landry Stéphane Baki, Aser Minoungou, Raffaella Ravinetto

**Affiliations:** a Department of Public Health, Institute of Tropical Medicine, Antwerp, Belgium; b Health Products Division, World Health Organization (WHO), Geneva, Switzerland; c Independent Consultant, Noirmoutier-en-l'île, France; d Central Medical Stores for Essential Medicines and Medical Equipment in Madagasar, SALAMA, Centrale d'Achats de Médicaments Essentiels et Materiel Médical de Madagascar, Antananarivo, Madagascar; e Benin Health Products Supply Society, SoBAPS, Société Béninoise pour l'Approvisionnement en Produits de Santé, Cotonou, Benin; f New Public Health Pharmacy in Cote d'Ivoire, NPSP, Nouvelle Pharmacie de la Santé Publique de Côte d'Ivoire, Abidjan, Côte d'Ivoire; g Directorate of Pharmaceutical Activities, DAP, Direction de l'Activité Pharmaceutique, Ministry of Health, Public Hygiene, and Universal Health Coverage, Abidjan, Côte d'Ivoire; h Central Medical Stores for Essential Generic Medicines and Medical Supplies in Burkina Faso, CAMEG Centrale d'achat des médicaments essentiels génériques et des consommables médicaux, Ouagadougou, Burkina Faso; i African Association of Central Medical Stores for Essential Medicines, ACAME, Association des Centrales d'Achats de Médicaments Essentiels, Ouagadougou, Burkina Faso; j School of Public Health, University of the Western Cape, Cape Town, South Africa

**Keywords:** Essential medicines, supply, price, access, public sector, pharmaceutical policies, Africa

## Abstract

The availability and affordability of essential medicines and other health products are critical to building resilient health systems and achieving Universal Health Coverage (UHC). In many Francophone African countries, Central Medical Stores (CMS) play a key role in procuring and distributing medicines for the public sector. As such, they generate first-hand data and knowledge on availability, pricing, and the performance of national supply chains. This Comment argues that CMS should be recognised not only as technical providers, but as strategic partners in national pharmaceutical policy and health system governance. Despite their constraints and the challenging environments in which they operate, CMS routinely generate supply-chain data that often remain underused beyond day-to-day operations. Where existing data sources on access to medicines are incomplete or unavailable, CMS data can help fill key gaps and inform planning, monitoring, and policy decisions. To operationalise this shift, we propose strengthening CMS analytical and policy-interface capacity and formalising collaboration and governance mechanisms with Ministries of Health and partners.

## Introduction

The availability and affordability of essential medicines and other health products are critical drivers to build resilient national health systems and to achieve Universal Health Coverage (UHC) (Wirtz et al., 2017). Yet data on access to medicines in global agendas – from the Millennium Development Goals to the Sustainable Development Goals – remain limited. Agreed measures of access have proven complex to operationalise, while experts emphasise that progress should build on existing data sources (Gotham et al., 2016; Jenei & Wirtz, 2024).

In Francophone Africa, procurement and distribution of medicines for the public sector are typically centralised through Central Medical Stores (CMS) (Seiter, 2010). These institutions hold historical but generally unpublished data on volumes and prices of medicines. While not sufficient to capture the full picture of access, such data could help to inform trends in consumption, barriers to availability and affordability, and access gaps for specific conditions. Beyond supply management, CMS information could contribute to analyse broader patterns, for example, antibiotic use, demand for mental health medicines, or rising needs related to pathologies associated with climate change.

When policymakers, donors, and other stakeholders underuse CMS information, the impact extends beyond logistics to financing, priority-setting, and health system strengthening. Consequences include misaligned budgeting and costing (for example for essential medicines and benefits packages), procurement regulations and practices that are not fit for purpose, missed opportunities to detect recurrent access bottlenecks and inequities, and delayed policy and programme responses during disruptions or donor transitions.

This comment is inspired by experiences shared during a webinar held on 6 November 2024 (Is access to medicines improving in Francophone Africa? What CMS know that no one asks, 2024), and by exchanges among participants and organisers. The webinar brought together speakers from four African countries representing Central Medical Stores, Ministries of Health, and WHO, and engaged around 100 participants. We argue that the information and knowledge CMS generate – despite gaps and limitations – remain an underused resource.

What this Comment adds is a shift in perspective: it argues for recognising CMS as strategic institutional partners in the health system architecture, and outlines governance arrangements needed to translate their expertise and routine information into system-level decisions.

## The CMS mission confronted with old and new challenges

CMS are government-established agencies responsible for ensuring the supply of medicines and health products to healthcare facilities in the public sector (Seiter, 2010). Over time, CMS have adopted diverse status, governance and operational models, ranging from traditional public institutions to business-oriented autonomous structures, shaped over time by reforms and evolving policies (Blaise et al., 1998; Watson et al., 2013). Some CMS hold a monopoly over public sector supply, while others coexist with alternative procurement channels (Direction de la Pharmacie, des Laboratoires et de la Médecine Traditionnelle, 2024). Despite differences, all CMS share a core mission: to ensure the accessibility of affordable, quality-assured essential medical products for the public health sector.

CMS face numerous challenges in fulfilling this mandate, which can be broadly categorised into three areas.

### Financial fragility and dependence on global markets

National governments across all income levels struggle, with the inherent complexity of sourcing and procuring medicines through international supply chains (Chapman et al., 2022; Gray & Manasse, 2012). Dependence on global suppliers is a well-recognised driver of delays and shortages (Chapman et al., 2022). Yet CMS operate in an even more constrained environment. They are expected to function as self-sustaining entities, generating revenue through medicine sales to cover operational costs. In practice, however, they serve poor populations, and must cope with sharp fluctuations in medicine prices, often in contexts where pricing policies are weak or absent (Govindaraj et al., n.d., p. 5165; World Health Organization, 2010).

Government subsidies and donor support can ease financial constraints, but many CMS depend heavily on short-term loans to maintain operations and supply continuity (Direction de la Pharmacie, des Laboratoires et de la Médecine Traditionnelle, 2024). In countries lacking health insurance systems, financial flows are further destabilised by reliance on cost recovery mechanisms, based on patients’ out-of-pocket payments (Towards universal health coverage in the WHO African Region: tracking financial protection, 2024). This fragility and unpredictability of financial flows are compounded when CMS must supply medicines on credit, which is frequently the case (Direction de la Pharmacie, des Laboratoires et de la Médecine Traditionnelle, 2024).

The financial volatility undermines procurement capacity. An effective participation in international markets requires, besides business intelligence and knowledge of supplier landscapes, strong cash positions to negotiate prices, secure stock, and maintain credibility with suppliers – even more during emergencies (Central Medical Stores Trust, 2024; Musuva et al., 2024).

### A need to strengthen capacity and align with global health agendas

In the 2000s, the fragility of national supply systems emerged as an important threat to global health initiatives targeting HIV, tuberculosis, malaria, reproductive health, and vaccination (Millot G, 2006). In response, new internal and external investments enabled several CMS in Africa to upgrade infrastructure, equipment, management, operations, and information systems, for improving efficiency and aligning with international quality standards (Mabirizi et al., 2018; Supply Chain Management Systems, 2015; Systems for Improved Access to Pharmaceuticals and Services, 2018). In this context, the African Association of Central Medical Stores (ACAME), which brings together most CMS in Francophone Africa, has been playing a key role in fostering progress and cross-country collaboration. Working closely with the World Health Organization (WHO), ACAME has coordinated multiple donor-funded initiatives, to strengthen technical capacity, advance quality assurance, and promote best practices across member institutions (Ideas and impact, 2016; USAID MTaPS Program, 2024).

### Global crises and rising expectations

The progress made by CMS is continuously challenged by persistent difficulties, global uncertainties, and rising expectations. The growing impact of force majeure events – such as the COVID-19 pandemic and geopolitical conflicts (Tirivangani et al., 2021) – exacerbated supply disruptions and drove up medicine prices (Barber, 2024). Armed conflicts and climate change cause population displacement, resulting in more frequent outbreaks and fuelling antimicrobial resistance (Pallett et al., 2023). All these factors increase the demand for medical supplies, while making last-mile delivery more difficult and costly.

Even in non-conflict settings, medical needs are increasing due to aging populations, higher demand for NCD treatments, and health systems expanding coverage to innovative prevention and treatment. These *per se* positive changes require CMS to continuously adapt to the evolving environment, generally in the absence of additional or innovative funding mechanisms (Omotoso et al., 2023).

## The unique value of CMS data on health systems

CMS have a unique insider knowledge of the global pharmaceutical market, gained from their central role in procuring medical products. In addition, they manage extensive logistics and financial data through their operations. Most African CMS have adopted integrated information systems – i.e. Enterprise Resource Planning (ERP) – to handle the complex daily financial and supply chain activities, spanning thousands of products and clients. In some countries, these systems are linked to the data reported from health facilities, serving as important interfaces within the broader logistics information system (Mabirizi et al., 2018).

In typical private businesses, such data are proprietary and primarily used for internal operations and planning, such as accounting and warehouse management. CMS also rely on these data for internal management, but in addition, they report to Ministries of Health and donors, providing key indicators to monitor supply chains for medicine groups such as HIV, tuberculosis, and malaria programmes (Defawe, 2015; Direction de l’Activité Pharmaceutique, 2024; Mabirizi et al., 2018). Additionally, CMS are frequently engaged by Ministries of Health and partners to lead or support forecasting and procurement planning for donor-funded programmes (Direction de l’Activité Pharmaceutique, 2024; Programme National de l’Activité Pharmaceutique, 2018).

Beyond forecasting and procurement planning, CMS data can offer governments and partners valuable insights into medicine consumption trends, regional variations, and price changes. In countries where CMS are better integrated into national health strategies, these data have informed broader planning and monitoring efforts (Direction de l’Activité Pharmaceutique, 2024). If systematically analysed and linked to national planning tools, CMS data could help Ministries of Health adjust procurement budgets, align supply chains with benefits package design, and develop early warning indicators of potential supply chain or service delivery disruptions. For example, volume and price data by product and over time can inform realistic costing of health benefits packages, while availability indicators can provide a useful proxy for tracking the evolution of access to medicines in the public sector.

Yet such applications remain rare, and despite their potential to inform health financing and policy, CMS data have important limitations that must be acknowledged. First, CMS work with a predefined list of products, guided by national policies such as the Essential Medicines List, and constrained by their own procurement capacity. Thus, their data may not capture all medicines or formulations needed by the population, creating gaps in availability (Direction de l’Activité Pharmaceutique, 2024). Second, CMS supply planning relies on demand and distribution data, which may not accurately reflect actual needs, especially when health facilities procure from multiple sources. Limited planning capacity and financial constraints at the facility level can cause inconsistent orders and misalignment with real needs, while inadequate prescribing practices may further distort demand data (Aderaw Yenet et al., 2023; Kaupa et al., 2021; Ravinetto et al., 2024; Shukar et al., 2021).

Despite the systemic inefficiencies that may affect their operations, CMS occupy a central position at the crossroads of public service and market-driven supply systems. They hold strategic insights into healthcare needs, supply challenges, and overall health system performance. Despite constraints due to a combination of insufficient and unpredictable financing, operational inefficiencies, and pressure to respond to national and global health demands, CMS remain essential actors in the effort to achieve equitable access to medicines and other health products.

## Conclusions

As a Comment informed by experiences shared during a public webinar and follow-up exchanges, and complemented by existing literature, we propose concrete recommendations to enable the strategic use of CMS expertise and the information they generate.

The challenges CMS face – ranging from financing constraints and fragile supply chains to inadequate pricing policies and unpredictable demand – have direct implications for the resilience and responsiveness of national health systems. Yet these same challenges also highlight the value of CMS as strategic partners: beyond their logistical role, they hold critical networks, knowledge, and data that can inform operational research, monitoring and evaluation, decision-making, and policy development.

To fully leverage their role, CMS should be recognised not only as technical service providers, but as institutional stakeholders in national health policy. First, they *are* critical operational actors in the supply chain, thanks to the management of financial and logistics data, procurement expertise, and real-time knowledge of medicine flows. Second, they *should* actively participate in the development of national evidence-based strategies to improve access to medicines and advance UHC.

Realising this potential requires a shift in perspective, which should be supported by sustained investment, closer collaboration with Ministries of Health, national regulators, and researchers, and meaningful integration into national health and pharmaceutical systems. Here, we suggest four action lines. First, CMS themselves may need to adopt a broader institutional mindset: for example, by designating dedicated focal points within their staff who can liaise with Ministries of Health, translate operational lessons into public health policies, and provide advice based on robust data analysis. Second, Ministries of Health should in turn establish reciprocal focal points and mechanisms to integrate CMS perspectives into health planning and policy decisions. Third, MOH and CMS may need to jointly clarify and formalise their respective roles and responsibilities, so that CMS’ advisory function is recognised and valued, while limiting possible conflicts of interest between their respective supply and policy-support roles. Finally, donors and technical partners can support this transition by funding capacity-building for CMS in data analysis and policy engagement, and by promoting governance arrangements that integrate CMS contributions into the broader pharmaceutical and health system strategies.
